# Designing next-generation platforms for evaluating scientific output: what scientists can learn from the social web

**DOI:** 10.3389/fncom.2012.00072

**Published:** 2012-10-01

**Authors:** Tal Yarkoni

**Affiliations:** Institute of Cognitive Science, University of Colorado BoulderBoulder, CO, USA

**Keywords:** data sharing, open access, peer review, publishing, scientific evaluation

## Abstract

Traditional pre-publication peer review of scientific output is a slow, inefficient, and unreliable process. Efforts to replace or supplement traditional evaluation models with open evaluation platforms that leverage advances in information technology are slowly gaining traction, but remain in the early stages of design and implementation. Here I discuss a number of considerations relevant to the development of such platforms. I focus particular attention on three core elements that next-generation evaluation platforms should strive to emphasize, including (1) open and transparent access to accumulated evaluation data, (2) personalized and highly customizable performance metrics, and (3) appropriate short-term incentivization of the userbase. Because all of these elements have already been successfully implemented on a large scale in hundreds of existing social web applications, I argue that development of new scientific evaluation platforms should proceed largely by adapting existing techniques rather than engineering entirely new evaluation mechanisms. Successful implementation of open evaluation platforms has the potential to substantially advance both the pace and the quality of scientific publication and evaluation, and the scientific community has a vested interest in shifting toward such models as soon as possible.

Archimedes is widely considered one of the greatest mathematicians and scientists of antiquity. Yet he lived during a period of history (the third century BC) not known for meticulous record keeping, and our appreciation of his seminal contributions consequently depends largely on good fortune. Because of his correspondence with the scholars Conon and Dositheus at the library of Alexandria, we now know of his seminal contributions to geometry and mechanics—work that formed the basis of numerous engineering advances and mathematical discoveries in subsequent centuries (Heath, 1897; Chondros, 2010a,b). But any numbers of slight deviations in the course of history—say, a crucial letter lost at sea, or a librarian's decision to reuse one of Archimedes' palimpsests—could have resulted in the permanent loss of his seminal works (and indeed, a number have never been recovered). In Archimedes' time, and through most of modern human history, the rate of scientific and technological progress depended not just on who discovered what, but also on how good people were at preserving knowledge of what they discovered for future generations. And since record keeping was a difficult business that involved allocation of limited resources, progress also depended heavily on the scholarly community's collective ability to accurately determine *which* work was worth keeping around for posterity.

Modern technology has now solved the problem of preservation; contemporary scientists can rest assured that virtually every scientific article published today will exist in digital form in perpetuity. One might intuitively expect that this alleviation of the preservation bottleneck would also eliminate the selection problem; after all, if it costs virtually nothing to publish and preserve, why bother to suppress a scientific manuscript that could be useful to someone else down the line, however, improbable the odds? Yet in many respects, the scientific community still behaves as though record keeping were a difficult enterprise and paper a scarce commodity. We spend months waiting to hear back from reviewers at journals with 90% rejection rates, anguishing over the prospect that our work might not see the light of day, even though we could disseminate our manuscript to the whole world at any moment via the web. We rely heavily on a select few individuals to pass judgment on our work, even though dozens or hundreds of other researchers are likely to form an informed opinion of its merits within days of official publication. And while we wait for the reviews to come in, we silently fret over the possibility that we might be “scooped” by someone else, even though all it takes to establish scientific precedence is one timestamped upload in a preprint repository.

The continued reliance on an anachronistic publication and evaluation model is striking given the widespread awareness of its many limitations (Smith, 2006; Casati et al., 2007; Jefferson et al., 2007; Young et al., 2008; Ioannidis et al., 2010). Many scientists seem all too happy to move away from the current publishing model and adopt an alternative model that emphasizes open access and “crowdsourced” evaluation. But progress toward such a goal has been relatively slow. While preprint servers such as arXiv.org have attained near universal usage in some disciplines, such platforms provide few if any tools for evaluation of manuscripts. Conversely, the few platforms that do allow users to evaluate manuscripts post-publication (e.g., the Public Library of Science's platform; http://plos.org) have a restricted scope and limited userbase (e.g., analysis of publicly available usage statistics indicate that as of this writing, PLoS articles have received an average of 0.06 ratings and 0.15 comments; http://www.plosone.org/static/almInfo.action).

Understandably, norms take time to change; what's surprising is perhaps not that scientists still rely on publishing and evaluation models developed centuries ago, but that they do so in the face of available alternatives. While the scientific community has been slow to embrace emerging information technology, that technology has itself evolved very quickly, and now supports tens of thousands of websites featuring a prominent social component—what has come to be known as the *social web*. In many respects, the challenges faced by popular social web applications—spanning everything from Amazon to Netflix to reddit to Last.fm–closely resemble those involved in evaluating scientific work: How can we combine disparate ratings from people with very different backgrounds and interests into a single summary of an item's quality? How do we motivate users to engage with the platform and contribute their evaluations? What steps should we take to prevent people from gaming the system? And can we provide customized evaluations tailored to individual users rather than the userbase as a whole?

In the rest of this paper, I discuss a number of principles that should guide the implementation of novel platforms for evaluating scientific work. The overarching argument is that many of the problems scientists face have already been successfully addressed by social web applications, and developing next-generation platforms for scientific evaluations should be more a matter of adapting the best currently used approaches than of innovating entirely new ones (cf. Neylon and Wu, 2009; Priem and Hemminger, 2010). Indeed, virtually all of the suggestions I will make have, in one form or another, already been successfully implemented somewhere on the web—often in a great many places.

I begin by briefly reviewing the limitations of the current publishing and evaluation model. I argue that since a transition away from this model is inevitable, and is already in progress, it behooves us to give serious thought to the kinds of platforms we would like to see built in the near future—and increase our efforts to implement such platforms. I then spend the bulk of the article focusing on three general principles we should strive to realize: openness and transparency, customizability and personalization, and appropriate incentivization. Finally, I conclude with a consideration of some potential criticisms and concerns associated with the prospect of a wholesale change in the way the scientific community evaluates research output.

## Limitations of current practice

Although the focus of the present article and others in this collection is on constructive ideas for new scientific evaluation platforms rather than on critiques of existing models, a brief review of some major limitations of current evaluation practices will provide a useful backdrop for subsequent discussion of alternative approaches. These limitations include the following.

### Slowness and inefficiency

Most articles that eventually get published in peer-reviewed journals go through several cycles of revision and re-review—often at different journals. Typically, months or years elapse between the initial submission and official publication of a manuscript (Ray, 2000; Ellison, 2002; Hall and Wilcox, 2007; Kravitz and Baker, 2011). Most of that time is spent passively waiting rather than actively revising or reviewing; authors have to wait for editors, editors have to wait for the slowest reviewer, and when a paper is rejected, everyone has to wait for the authors to revise and resubmit the manuscript to a different journal. There's no principled justification for such delays and inefficiencies; they simply fall out of current publishing models, with many journals having to reject the vast majority of submissions received in order to preserve a reputation for quality and selectivity. Improving the speed and efficiency of the review process could potentially have a dramatic impact on the rate of scientific progress.

### Opacity

Because the peer review process is typically conducted behind closed doors, most reviews leave no cumulative record for other scientists to peruse, and allow no independent evaluation of the reviews or reviewers themselves. The problem with lack of transparency is that the quality of reviews is highly variable, frequently leading to rejection of articles for spurious reasons (see below). Unfortunately, under the current model, consumers have no way to evaluate the process that led up to a final decision, or to review any of the interactions between authors, reviewers, and editors. This opacity increases the likelihood of incorrect judgments about a paper's merits, and runs completely counter to the cumulative and open nature of the scientific enterprise. If we don't know who said what about a manuscript and how the manuscript's authors responded, we run a high risk of overlooking or repeating potentially important mistakes.

### Low reliability

Current evaluation practices might be defensible if there were empirical evidence that such practices achieve their goals; but formal studies consistently suggest that conventional pre-publication peer review is of limited utility in establishing the quality of manuscripts (though it is undeniably better than no peer review at all). A recent random-effects meta-analysis of 48 studies, comprising 19,443 manuscripts, estimated an inter-rater intra-class correlation of only 0.34 (Bornmann et al., 2010). Since most articles are evaluated by only two or three reviewers prior to publication, and editorial decisions typically follow those of reviewers, it follows that many decisions to accept or reject a manuscript are not appreciably better than chance. This point is corroborated by the grossly uneven distribution of citation rates for articles published in top journals such as Nature and Science (which explicitly select articles on the basis of perceived impact): a minority of articles typically account for the vast majority of citations, and a sizeable proportion of published articles receive few or no citations (Seglen, 1997; Dong et al., 2005; Mayor, 2010). While citation counts are not a direct measure of paper quality, there is little reason to suppose that journal impact factor predicts other metrics or expert judgments any better. To the contrary, retrospective evaluations have found modest or no correlations between journal impact factor and expert ratings of impact or quality (Bath et al., 1998; West and McIlwaine, 2002; Maier, 2006; Sutherland et al., 2011). Such findings imply that the heavy emphasis scientists often place on “high-impact” publications when evaluating other researchers' work is likely to be misplaced.

### Lack of incentives

Reviewing scientific manuscripts is time-consuming and effortful. Unfortunately, peer reviewers have relatively little incentive to do a good job. Outside of a sense of duty to one's profession and peers, and perhaps a pragmatic desire to curry favor with editors, scientists have little to gain by volunteering their time as reviewers, let alone by turning in high-quality reviews on time (Mahoney, 1977; Hojat et al., 2003). Indeed, in some cases, reviewers may even have incentives to write *bad* reviews—for instance, when a researcher is asked to evaluate a competitor's manuscript. There's no doubt that the vast majority of scientists will do the right thing in such cases; but it surely seems like bad policy to rely on a system that depends almost entirely on communal goodwill. An ideal evaluation model would directly incentivize the behaviors that maximize the success of the scientific enterprise as a whole, and conversely, would actively deter those that threaten the quality or efficiency of that enterprise.

### A transition is inevitable

The limitations reviewed above exist for good reasons, of course. But those reasons are almost entirely historical. When papers were published exclusively in print and scientific communication took place via the postal service, it made sense to restrict publication to a minority of papers that passed some perceived litmus test for quality. But such constraints don't apply in an age of electronic communication, open access repositories, and collaborative filtering algorithms. Now that the marginal cost of replicating and disseminating manuscripts has dropped to essentially nothing, it makes little sense to artificially restrict the availability or flow of scientific information. There's a continued need for quality control, of course; but that can be achieved using “soft” filtering approaches that dynamically emphasize or deemphasize information *ad hoc*. It doesn't require destructive approaches that permanently remove a large part of relevant data from the record. If Archimedes in his day had had the option of instantly depositing his work in arXiv, it's doubtful that anyone today would accuse him of wasting a few bytes. It's relatively easy to ignore information we don't need, but not so easy to recreate information that no longer exists.

One might argue that flooding the scientific literature with papers that have received little or no prior scrutiny would result in information overload and make it impossible to separate good research from bad. But whatever the merit of this argument (and I argue below that it has little), it seems clear at this point that the ship has already sailed. With a modest amount of persistence, scientists can now place virtually any manuscript in a peer-reviewed journal somewhere (Chew, 1991; Ray, 2000; Hall and Wilcox, 2007)—and often in well-respected venues. For instance, PLoS ONE, the world's largest journal, published over 7000 articles in 2010, spanning nearly all domains of science, and accepted approximately 70% of all submissions (http://www.plosone.org/static/review.action). This model appears so financially successful that Nature Publishing Group and SAGE have both recently launched their own competing open-access, broad-scope journals (Scientific Reports and SAGE Open). To put it bluntly, between megajournals like PLoS ONE and thousands of specialized second- and third-tier journals, we already *are* publishing virtually everything. But we're doing it very slowly and inefficiently. So the real question is no longer whether or not the scientific community should transition to an open publishing model (Harnad, 1999; Shadbolt et al., 2006); it's how to handle the inevitable flood of information most efficiently and productively. Our current approach is to rely on heuristics of dubious value—e.g., journal impact factors. But there are far better technological solutions available. The rest of this article discusses a series of principles scientists should strive to respect when implementing new platform and that have already been implemented with great success in many social web applications that face similar evaluation challenges.

## Openness and transparency

To combat the opacity of the current peer review system, openness and transparency should be central design features of any next-generation scientific evaluation platform. In this context, openness doesn't just mean making reviews of papers accessible online; it implies a fundamental level of transparency and data accessibility that should reside at the very core of new platforms. Multiple layers of information—including nearly all the data amassed by that platform over time—should be freely available and programmatically accessible to interested parties.

### Open access to (nearly) all content

Arguably the single most important desideratum for a next-generation evaluation platform is providing open access to the reviews, comments, and ratings of manuscripts generated at all stages of the evaluation process. Setting aside for the moment the question of whether reviewers should be forced to disclose their identities (see below), there is little reason to withhold the content of reviews and ratings from the public—at least in aggregate form (e.g., providing the mean ra ting of each manuscript). Making evaluations openly accessible would have several substantial benefits. First, it would allow researchers to evaluate the evaluators; that is, researchers would be able to determine the quality of the reviews that influence the reception of an article, and adjust that reception accordingly. Unscrupulous researchers would, for instance, no longer have the power to reject competitors' work by providing excessively negative reviews, since those reviews would themselves be subject to evaluation. Second, when implemented on a sufficiently large-scale, an open database of evaluations would provide a centralized forum for discussion of scientific work, which currently occurs in a piecemeal and much less efficient fashion elsewhere online and offline. Third, open access to reviews would allow researchers to receive credit for evaluating others' work, and hence provide greater incentive to participate in peer review.

All three of these principles are already embodied in many existing community-oriented websites. One particularly effective example is implemented on the popular social news website reddit (reddit.com), which features threaded conversations that allow users to comment and vote on both original submissions and other users' comments. Submissions and comments can then be sorted in a variety of ways (e.g., by top score, novelty, by amount of controversy, etc.). The result is a highly efficient collaborative filtering system (Schafer et al., 2007) that rapidly differentiates between high- and low-quality submissions. Moreover, the comments exert a strong influence on the reception of the original submissions; in many cases, an astute comment or two (e.g., when critical questions are raised about the veracity of information provided in a link) leads to rapid adjustment of a submission's score. And since comments are themselves subject to evaluation, the process is iterative and encourages genuine discussion between users with differing opinions. The net result is an openly accessible record of (mostly) intelligent debate over everything from YouTube videos to government bills to old photographs. The same type of open discussion model could potentially greatly facilitate evaluation of scientific manuscripts.

### Transparent identities

While there appear to be few downsides to making the *content* of reviews and ratings openly and easily accessible within a post-publication framework, the question of whether to force disclosure of reviewers' identities is a more delicate one. There's a common perception that peer reviewers would refuse to review papers if forced to disclose their identities, and that anonymous reviews are a necessary evil if we want researchers to express their true views about manuscripts (Fabiato, 1994; Ware, 2007; Baggs et al., 2008). This perception appears to be unfounded inasmuch as empirical studies suggest that forcing reviewers to disclose their identities to authors and/or readers only modestly increases refusal rates while improving the tone of reviews and leaving their overall quality unaltered (Justice et al., 1998; van Rooyen et al., 1998; Walsh et al., 2000; van Rooyen et al., 2010). Moreover, one can legitimately question whether anonymity currently allows reviewers to go to the opposite extreme, expressing excessively negative or unfair views that the light of day might otherwise moderate.

Nonetheless, privacy concerns deserve to be taken seriously. We can distinguish between technical and sociological questions related to identity disclosure. From a technical standpoint, the principle is clear: any evaluation platform should build in tools that allow users a range of privacy management options, ranging from full disclosure of identity (including real names, institutional affiliations, etc.) to pseudonymous or entirely anonymous posting. The sociological question will then arise as to how much transparency of identity is desirable, and how to best motivate that degree of disclosure. A strong case can be made that some data should remain private by default (except in the aggregate); for instance, it would probably be a bad idea to force public display of users' ratings of individual articles. While greater transparency may generally be a good thing, we shouldn't let the perfect be an enemy of the good: if the only way to encourage widespread adoption of a next-generation evaluation platform is to allow pseudonymity or anonymity that seems preferable to building an idealistic platform that no one wants to use. And as I discuss in more detail below, there is good reason to believe that given a well-structured reputation management system, most users would voluntarily opt to disclose their identities.

### Public APIs

Application programming interfaces (APIs) play a central role in modern web applications. Public APIs allow third-party developers and users to plot custom bicycle routes on Google Maps, to “mashup” different YouTube videos, and to integrate Twitter streams into their own websites and applications. API-based access to the data generated by a successful scientific evaluation platform would facilitate the development of novel third-party applications, in turn spurring greater adoption of a platform and promoting further innovation. Given a platform that aggregates citation data, ratings, reviews, and comments for every paper in PubMed, and makes such data accessible via API, third party developers could build a broad range of applications—for instance, article recommendation tools (“users who liked this paper also liked these ones…”), specialized aggregators that selectively highlight a subset of articles defined by some common interest, and customizable evaluation metrics that allow users to generate their own weighting schemes for quantitative assessment of articles, journals, researchers, or institutions.

Although the deployment and adoption of research-related APIs is still in early stages, several services have already begun to provide public API access to their data. Notable examples are the Public Library of Science (PLoS) API (http://api.plos.org), which provides access to article-level metrics (e.g., page views and downloads) for tens of thousands of PLoS articles, and the Mendeley API (http://dev.mendeley.com), which provides programmatic access to a crowdsourced research database of over 100 million articles and growing. An explicit goal of these APIs—and in the case of Mendeley, of an accompanying release of usage data for nearly 5 million papers (http://dev.mendeley.com/datachallenge)—is to support development of new research tools such as article recommendation systems (discussed in the next section). These releases represent only the beginning of what promises to be a deluge of publicly accessible data relevant to the evaluation of scientific output.

## Personalization and customizability

There was a time not too long ago when people decided what movies to watch, or what music to listen to, largely on the basis of consensus opinion and/or the authoritative recommendation of a third party. While such factors still play an important role in our choice of media, they have, in many cases, been superseded by social web applications explicitly designed to provide personalized recommendations based on each individual's prior history and preferences. Sophisticated recommendation systems at the heart of many of the web's most popular sites (e.g., Netflix, Amazon, Last.fm, and Google News) now provide nearly effortless ways to identify new products and services we (as opposed to other people) are likely to enjoy (for review, see Adomavicius and Tuzhilin, 2005; Pazzani and Billsus, 2007; Schafer et al., 2007). The revolutionary impact of such systems lies in their recognition that what people predominantly care about is how much *they* like a product. Other people's evaluations, while informative, are generally helpful only to the extent that they provide a reasonable proxy for one's own preferences.

Broadly speaking, recommendation systems come in two flavors. *Collaborative filtering* approaches rely on user-provided ratings to generate recommendations (Schafer et al., 2007). Make a few 5-point ratings on Netflix, and you'll start receiving suggestions for movies that similar users liked; view a product on Amazon, and it'll try to sell you related products others have bought. *Content-based* approaches rely on objective coding of different aspects of a product or service in order to identify similar items (Pazzani and Billsus, 2007). For example, the Pandora music service bases its recommendations on expert ratings of hundreds of thousands of songs (Casey et al., 2008). Empirical studies demonstrate that both collaborative filtering and content-based recommendation systems—as well as many hybrid approaches—are capable of accurately predicting user preferences across a broad range of domains, including commercial products (Pathak et al., 2010; Sarwar et al., 2000), movies (Miller et al., 2003; Bennett and Lanning, 2007), news articles (Phelan et al., 2009; Liu et al., 2010), leisure activities (Ducheneaut et al., 2009), and musical tastes (Yoshii et al., 2008; Barrington et al., 2009).

In principle, the scientific community could use similar filtering approaches to evaluate scientific output. The fundamental challenge time-pressed researcher's face when evaluating the scientific literature closely resembles the one that consumers in other domains face—namely, how to filter an unmanageable amount of information down to only those items that are likely to be of substantive interest. Currently, scientists address this problem using heuristics of varying quality, e.g., by focusing on highly-cited papers that appear in prestigious journals, signing up for keyword alerts, performing targeted literature searches, and so on. Such approaches can work well, but they're time consuming and effortful. Recommendation systems offer what is, in principle, a superior alternative: instead of requiring explicit effort to identify items of potential interest, the system continuously mines an accumulated database of article metadata and user ratings to generate recommendations. Preliminary efforts using content-based (Dumais and Nielsen, 1992; Basu et al., 2011), collaborative filtering (Bogers and Van Den Bosch, 2008; Naak et al., 2009), or hybrid (Torres et al., 2004; Gipp et al., 2009) approaches demonstrate the viability of automatically generating article recommendations. However, to date, such efforts have been conducted on a small scale, and lack an online, publicly accessible implementation with sufficient appeal to attract a critical mass of users. Developing an integrated recommendation system should thus be a major design goal of next-generation scientific evaluation platforms.

A successfully implemented article recommendation system would reduce researchers' reliance on other heuristics of debatable utility; for instance, given a system that could accurately predict which articles a user would find relevant and of high quality, there would be less need to focus attention on the journals in which articles were published. The goal of such recommendation systems wouldn't be to serve as final arbiter of the quality of new publications, but simply to filter the literature to a sufficient degree that researchers could efficiently finish the job. Moreover, as discussed in the next section, the presence of a recommendation system would provide a valuable incentive for users to contribute their own evaluations and ratings, enabling an evaluation platform to grow much more rapidly. Naturally, new concerns would arise during the course of implementation; for example, a recommendation system that attempts to identify papers that users will like risks creating an “echo chamber” where researchers only receive recommendations for papers that concord with their existing views (Massa and Avesani, 2007). However, such challenges should generally have straightforward technical solutions. For example, the echo chamber effect could be combated by limiting the weighting of users' favorability ratings relative to other criteria such as relevance of content, methodological rigor (as assessed by the entire userbase), and so on.

A second benefit that highly centralized, open access evaluation platforms would afford is the ability to develop customizable new metrics quantifying aspects of scientific performance that are currently assessed primarily subjectively. Consider, for instance, the task that confronts academic hiring committees charged with selecting a candidate from among dozens or hundreds of potential applicants. Since few if any committee members are likely to have much expertise in any given applicant's exact area of research, hiring decisions are likely to depend on a complex and largely subjective blend of factors. Is an applicant's work well respected by established people in the same field? Does she consistently produce high-quality work, or are many of her contributions incremental and designed to pad her CV? Does a middling citation rate reflect average work, influential work in a small field, or poor work in a large field? Is the applicant's work innovative and risky, or cautious and methodical?

Current metrics don't answer such questions very well. But a centralized and automated evaluation platform could support much more sophisticated quantitative assessment. For instance, a researcher's reputation among his or her peers could be directly quantified using explicit reputation systems (discussed in the next section) based on thousands of data points rather than three self-selected letters of recommendation. The novelty or distinctiveness of a researcher's individual publications could be assessed using algorithms that evaluate similarity of content across articles, pattern of citations to and from other articles, co-authorship, etc., thereby counteracting the pressure many scientists feel to maximize publication rate even if it results in redundant publications (Broad, 1981; Jefferson, 1998; Von Elm et al., 2004). The relative strengths and weaknesses of a research program could be measured by aggregating over users' dimensional ratings of innovation, methodological rigor, clarity, etc. And all of these metrics could be easily normalized to an appropriate reference sample by automatically selecting other authors in the system who works in similar content areas.

Developing an array of such metrics would be an ambitious project, of course, and might be beyond the capacity of any single organization given that funding for such a venture seems likely to come primarily from the public sector. But the public availability of rich APIs would off-load much of the workload onto motivated third parties. The recent proliferation of metrics such as the h-index (Hirsch, 2005), g-index (Egghe, 2006), m-index (Bornmann et al., 2008), and dozens of other variants (Bornmann et al., 2011) is a clear indicator that a large market exists for better measures of research performance. But such metrics are currently based almost entirely on citation counts; developing a centralized and open platform that supports much richer forms of evaluation (votes, ratings, reviews, etc.) seems likely to spur a broader revolution in bibliometrics (cf. Neylon and Wu, 2009; Lane, 2010; Priem and Hemminger, 2010).

In the longer term, the development of a broad range of evaluation metrics could lead to sophisticated new weighting schemes optimized for highly specific evaluation purposes. Instead of relying solely on recommendation systems to identify relevant articles, researchers would be able to explicitly manipulate the algorithms that generate summary evaluations of both individual articles and researchers' entire output. For instance, a hiring committee could decide to emphasize metrics assessing innovation and creativity over methodological rigor, or vice versa. An editorial board at a general interest journal could use metrics quantifying breadth of interest (e.g., diffusion of positive ratings across researchers from different fields) to select preprints for “official” publication. Science journalists could preferentially weight novelty when selecting work to report on. The degree of customization would be limited only by the sophistication of the underlying algorithms and the breadth of the available research metrics.

Providing a high degree of personalization and customizability wouldn't completely eliminate subjective criteria from evaluation decisions, of course—nor should it. But it would minimize the intensive effort researchers currently invest in filtering the literature and identifying relevant studies; it would reduce reliance on evaluation heuristics of questionable utility (e.g., identifying the quality of papers with the impact factor of journals); and it would provide objective bases for decisions that currently rely largely on subjective criteria. In view of the low reliability of classic peer review, and the pervasive finding that trained human experts are almost invariably outperformed by relatively simple actuarial models (Dawes et al., 1989; White, 2006; Hanson and Morton-Bourgon, 2009), we have every reason to believe that increasing the level of automation and quantitative measurement in the evaluation process will pay large dividends. And there is little to lose, since researchers would always remain free to fall back on conventional metrics such as citation rates if they so desired.

## Providing appropriate incentives

Suppose one implemented a platform with features such as those described in the preceding sections. Would scientists rush to use it? Would the database quickly fill up with lengthy reviews and deep comment threads? Probably not. Technical innovation is only one part of any novel publishing platform—and arguably not the most important part. New tools and platforms are often adopted quite slowly, even when they offer significant technical advantages over previous approaches. Users signing up for a service are generally not interested in what the service *could* be like in five years given widespread adoption; they're interested in the benefits they can obtain from the service if they start using it *right now*.

Many technically advanced platforms that could in principle enhance scientific communication and evaluation fail to appropriately incentivize their potential userbase. Consider the PLoS platform (http://plos.org), which has long enabled users to rate and review papers, with the goal of encouraging interaction between readers and/or authors. In theory, such a platform offers substantial benefits to the scientific community. If everyone used it regularly, it would be very easy to tell what other people—including leading experts in the field—thought about any given article. Unfortunately, the PLoS platform provides virtually no incentive to participate, and may even offer disincentives (Neylon and Wu, 2009; Nielsen, 2009). At present, if I spend an hour or two writing a critical review of a paper and sign it with my real name, very few people are likely to read my commentary—and those who do may well wonder why I'm wasting my time writing lengthy reviews on open access websites when I could be working on my own papers. As a consequence, only a small proportion of PLoS articles have received any comments, and a similar lack of engagement characterizes most other publishing platforms that provide a facility for online discussion of manuscripts (Neylon and Wu, 2009; Gotzsche et al., 2010).

Some critics have seized on the lack of community engagement as evidence of the flaws of a post-publication evaluation model (Poynder, 2011). But the reason that researchers haven't flocked to comment on PLoS articles seems very much like the reason editors often complain about how hard it is to find peer reviewers: there simply isn't any meaningful incentive to contribute. Getting researchers to invest their time building an online portfolio isn't only (or even primarily) about providing the *opportunity* to engage in online discussion; it's also about providing appropriate motivation.

As with many of the other problems discussed above, social web applications have already addressed—and arguably solved—the challenge of incentivizing a userbase to participate. Indeed, virtually every website that relies on user-generated product ratings and reviews faces much the same challenge. For instance, Netflix's business model depends partly on its ability to find you movies that you'll enjoy. That ability, in turn, depends on sophisticated quantitative modeling of movie ratings provided by Netflix users. Without the ratings, Netflix wouldn't be able to tell you that you're likely to enjoy *All About My Mother* if you enjoyed *Spirited Away*. But Netflix users don't rate movies out of an abiding respect for Netflix's bottom line; they rate movies so that Netflix can give them personalized movie recommendations. Netflix doesn't have to ask its users to behave charitably; it simply appeals directly to their self-interest. Analogous models are everywhere online: tell Last.fm or Grooveshark which songs you like, and they'll tailor the songs they play to your preferences; buy a product from Amazon, and it'll try to sell you related products others have bought; upvote a link on reddit and you get to exert direct (if weak) social influence on the community. Not only is the long-term goal—whether making money or building an online community—not emphasized on these websites; it's largely invisible.

*A priori*, it seems reasonable to expect the same type of model to work equally well for scientific evaluation. Many scientists decline invitations to review manuscripts because they can't spare a few hours on relatively thankless labor, but few scientists would be too busy to make a single 5-point rating after reading a paper—especially if it doing so helped the system recommend new papers. The long-term goal of creating a centralized platform for evaluation of scientific manuscripts wouldn't require much emphasis; done right, researchers would be happy to use the service simply as a recommendation engine or bibliography management tool. More sophisticated features (e.g., separate ratings along dimensions such as impact, innovation, and methodological rigor; threaded ratings and reviews of other reviews; etc.) could then be added incrementally without disrupting (and indeed, generally increasing) the appeal of the core platform.

Notably, at least one popular service—Mendeley (mendeley.com)—already appears to be taking precisely this kind of “passive” approach to community building. Initially billed as a web-based bibliography management tool, Mendeley recently introduced a public API that provides access to its data, and has already begun to add social networking features and statistical reports that could soon form the basis for a community driven recommendation system (http://dev.mendeley.com). Crucially, Mendeley has been able to grow its enormous crowdsourced database (over 1 million members and 100 million document uploads as of July, 2011) simply by providing an immediately valuable service, without ever having to appeal to its users' altruism. The success of this model demonstrates that the same principles that have worked wonders for commercial services like Netflix and Last.fm can be successfully adapted to the world of scientific evaluation. The challenge lies not so much in getting users to buy into long-term objectives that benefit the scientific community as a whole, but rather, in making sure that the short-term incentives that *do* drive initial user engagement are naturally aligned with those longer-term objectives.

### Reputation management

Providing short-term incentives such as personalized recommendations can help a platform get off the ground, but in the long run, building and maintaining an active community is likely to require additional incentives—ideally, the same ones that already drive scientific contributions offline. One prominent motivator is reputation. Currently, the primary mechanisms for building a reputation in most fields of science are tangible products such as journal publications, research grants, and conference presentations. Many other contributions that play essential roles in driving scientific progress—e.g., peer review, data sharing, and even informal conversation over drinks—historically haven't factored much into scientists' reputations, presumably because they've been difficult to track objectively. For instance, most scientific articles already include extensive discussion and evaluation of prior work—the quality of which bears directly on an author's reputation—but there is currently no way to formally track such embedded discussions and credit authors for particularly strong (or poor) evaluations. The development of new evaluation platforms will make it easy to quantitatively measure, and assign credit for, such contributions. The emerging challenge will be to ensure that such platforms also provide sufficient incentives for researchers to engage in desirable but historically underappreciated behaviors.

Here, again, scientists can learn from the social web. Reputation systems are at the core of many popular social web communities, including a number that cater explicitly to scientists. A common feature of such communities is that users can endorse or rate other users' contributions—e.g., indicating whether comments are helpful, whether product reviews are informative, and so on. A particularly relevant model is implemented on Stack Exchange (http://stackexchange.com), a network of over 50 question and answer sites geared toward professionals in different areas. While the most popular SE website (Stack Overflow) caters to software developers, the network also features a number of popular Q&A sites populated by academic researchers, including mathematics, statistics, physics, and cognitive science exchanges. A key feature of the SE platform is the use of a point-based reputation system. Users receive and award points for questions, answers, and edits that receive favorable ratings from other users. In addition to providing an index of each user's overall contribution to the site, users attain additional privileges as they gain reputation—e.g., the ability to promote, edit, or moderate others' questions. Thus, the system incentivizes users to participate in prosocial activities and penalizes unhelpful or low-quality contributions.

A notable feature of the SE platform is the explicit encouragement for users to post under their real names so as to leverage (and build) their offline reputations. This is most apparent on MathOverflow (http://mathoverflow.net), where many prominent users are tenured or tenure-track professors in mathematics-related fields at major research universities—many at the top of their fields. The success of this model demonstrates that, given the right incentives, even busy academics are willing to engage in online activities that, despite their obvious value to the community, previously weren't viewed as creditable scientific contributions. Consider a telling quote from a recent Simons Foundation article (Klarreich, 2011):

“I have felt the lure of the reputation points,” acknowledges Fields medalist Timothy Gowers, of Cambridge University. “It's sort of silly, but nevertheless I do get a nice warm feeling when my reputation goes up.”

Prior to the introduction of collaborative platforms like Stack Exchange, one might have been understandably skeptical of a famous mathematician revealing that he spends much of his time accumulating virtual points online (and as of this writing, Gowers ranks as one of the top 20 users on MathOverflow). But when the points in question are awarded for prosocial activities like asking and answering research questions, reviewing others' work, providing data, writing software, and giving advice, the scientific community stands to reap large benefits. Moreover, in addition to incentivizing prosocial contributions, SE-like reputation systems provide at least two other benefits. First, the reputation scores generated by platforms like Stack Overflow are themselves valuable in evaluating users' contribution to the scientific community, since a high reputation score by definition denotes a user who has made many positive contributions to the scientific community—mostly through channels that established metrics like citation counts don't adequately assess. Second, the ability to assign credit for contributions outside the traditional scope of scientific publication should incentivize contributions from many people who currently lack the means to contribute to science in more conventional ways. In particular, trained scientists who work at teaching positions or in non-academic settings would have a way of contributing in a meaningful and creditable way to the scientific enterprise even if they lack the time and resources to produce original research. Thus, carefully designed reputation systems stand to have a transformative effect on the communication and evaluation of scientific output.

## What happens to traditional pre-publication review?

Supposing new technological platforms do eventually transform the scientific evaluation process, an important outstanding question concerns the role of the traditional, journal-based evaluation model centered on pre-publication review. What happens to this model in a world populated by the kind of evaluation platforms envisioned here? Broadly speaking, there are two potential answers. First, one can envision hybrid evaluation models that combine the best elements of closed/pre-publication review and open/post-publication review. For example, one common argument in favor of pre-publication review is that it improves the quality of a manuscript prior to its public release (Goodman et al., 1994). Although the same benefit could arguably be provided by any platform that allows authors to continually revise their manuscript in response to post-publication reviews, one could certainly opt to retain an element of pre-publication review in an otherwise open platform. A straightforward way to implement such a system would be to grant authors permission over who can view a manuscript. In an initial “closed” period, authors would be free to invite selected peers to perform a closed review of the manuscript. The feedback received could then be used to revise the manuscript until the authors were satisfied. The key point is that the control over when to publish the “official” version of the manuscript would rest with the authors and not with an editor (though one might perhaps force authors to stipulate ahead of time whether or not each review would be made public, ensuring that authors could not suppress negative reviews *post hoc*). A major benefit of such an approach is that it would allow diligent authors to solicit feedback from competing (and likely critical) researchers, while penalizing less careful authors who rush to publish without soliciting feedback first. In contrast, under the current system, recommending critical reviewers is a risky and generally detrimental proposition.

The second way to answer the “what happens to conventional review” question is to admit that we don't really know—and, more importantly, that we don't really have to know. If conventional journals and pre-publication review play an indispensable role in the evaluation process, nothing much should change. Journals could go on serving exactly the same role they presently serve. All of the benefits of next-generation platforms discussed would apply strictly to post-publication review, after the standard review process has run its course (e.g., a deeply flawed article that happened to get by the peer review process at a top-tier journal would be susceptible to immediate and centralized post-publication critique). There would be no need to expend effort actively trying to eliminate conventional journals; a well-designed evaluation platform should be agnostic with respect to the venue (if any) in which manuscripts originally appear. Moreover, from a pragmatic standpoint, adoption of new post-publication evaluation platforms is likely to occur more rapidly if such platforms are presented as complements to conventional review rather than as competitors.

That said, it's easy to see how sophisticated post-publication evaluation platforms might ultimately obviate any need for conventional journals, and many commentators have argued that this is a perfectly logical and desirable end result (LaPorte et al., 1995; Odlyzko, 1995; Delamothe and Smith, 1999; Kingsley, 2007; Smith, 2010). Once it becomes clear that one can achieve efficient and reliable evaluation of one's manuscripts regardless of where (or whether) they're officially published, there will be little incentive for authors to pursue a traditional publication route. As a result, traditional journals may simply disappear over time. But the important point is that if this process happens, it will happen organically; nothing about the type of platform proposed here explicitly constrains the role of journals in any way. To the extent that traditional journals offer scientists an irreplaceable service, they will presumably continue to thrive. And if they don't offer a valuable service, we shouldn't mourn their passing.

## Putting it into practice

Having reviewed a number of basic design considerations, this section outlines one possible specification for a post-publication evaluation platform. In contrast to a number of recent proposals that focus on wholesale restructuring of the scientific publishing and evaluation process (e.g., Pöschl, 2010; Kravitz and Baker, 2011; Nosek and Bar-Anan, 2012), the platform described here focuses exclusively on facilitating centralized, publisher-independent post-publication review. The platform would exist independently of the existing pre-publication review system, and would not require articles to have undergone any prior form of peer review before being added to the system. Thus, there are effectively no major technical or legal barriers (e.g., copyright restrictions) to the immediate implementation of such a platform, and social barriers are also minimized by presenting the platform as a complement rather than competitor to traditional models.

**Figure 1:**
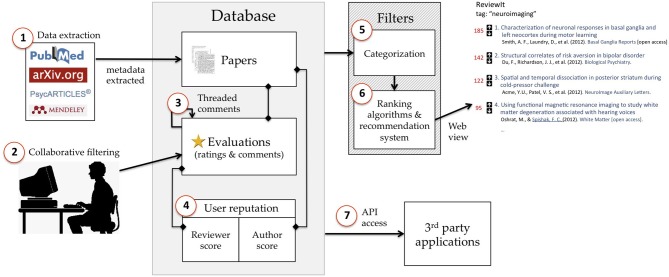
**Schematic illustration of key elements of the proposed model. (1)** Metadata for published or unpublished articles are automatically extracted from other sources and fed into the platform database. **(2)** Users have the ability to rate and/or comment on any article in the database. **(3)** Comments are threaded, allowing recursive evaluation. **(4)** A user's reputation reflects their aggregate contribution to the content in the database, with separate metrics for authorship and commenting. **(5)** Articles can be categorized into topics using both automated semantic classification techniques and manual curation. **(6)** Retrieved records are ranked based on a (potentially personalizable) combination of quality, relevance, and recency criteria. **(7)** Most of the data in the database can be accessed independently via API, allowing other parties to create their own evaluation-related applications. Numbered elements are described in greater detail in the main text.

A schematic of the proposed platform is provided in Figure 1. Given that the central argument of this paper is that most of the principles needed to establish a successful evaluation platform are already widely implemented on the social web, it should come as no surprise that the platform described here features few novel features—it's essentially a Reddit clone, with a few additional features borrowed from other platforms like Stack Exchange, Netflix, and Amazon. The platform features the following elements (corresponding to the circled numbers in Figure 1).

### Data extraction

The database is initially populated (and continuously updated) by pulling data from academic search engines and repositories. For example, many services like PubMed and ArXiv.org provide API access or free data dumps, ensuring that the evaluation platform can remain up to date without requiring any user input. The evaluation platform should link to all articles on the original publisher/repository website (when available), but should not take on the responsibility of facilitating access to articles. Articles that are currently behind a pay-wall would not become publicly accessible in virtue of having a discussion page on the evaluation platform website; the system would (at least initially) operate in parallel with the traditional publishing system rather than in competition.

### Collaborative filtering

**Figure 2:**
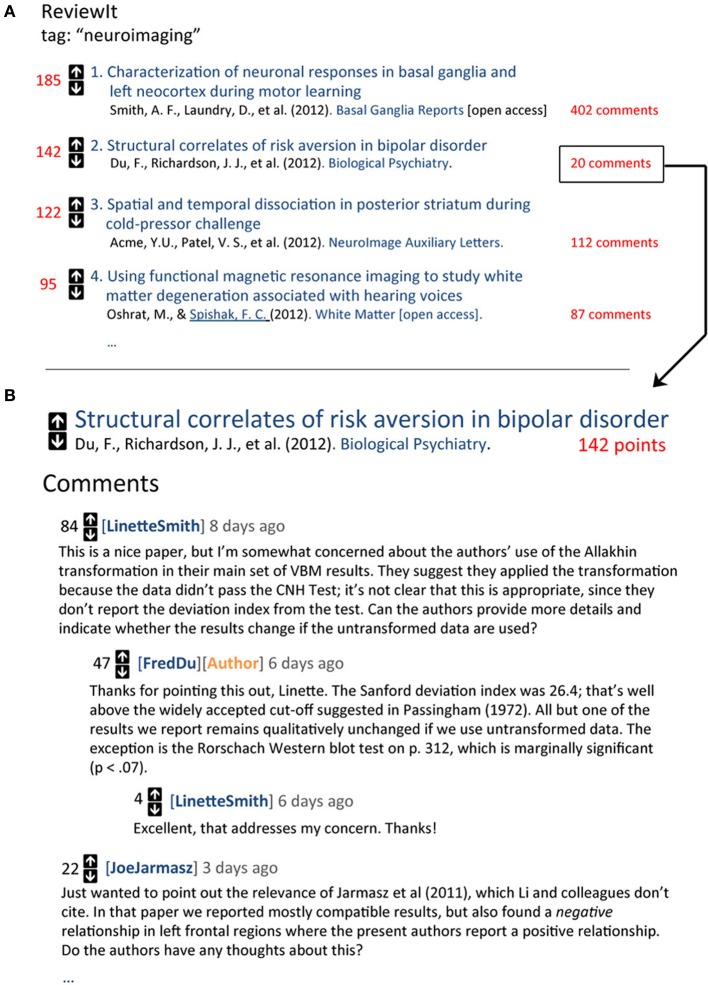
**Sample web views for a hypothetical “ReviewIt” post-publication evaluation platform modeled closely on reddit (http://reddit.com). (A)** Ranked listing of top articles tagged with the “neuroimaging” tag. Each record displays the current number of points (red), provides upvote/downvote arrows for rating the article, and displays basic information about the article (authors, journal, etc). **(B)** Clicking on an article's “comments” link takes the user to a discussion page where users can comment on any aspect of the article or respond to and rate other comments. Comments with high scores are displayed further up on the page, increasing their likelihood of influencing evaluation of the article.

At the core of the platform is a collaborative filtering approach that allows any registered user to rate or comment on any article in the database. These ratings and comments can then used to sort and rank articles and users in a variety of ways (see below). The simplest implementation would be a reddit-like voting system that allows users to upvote or downvote any article in the database with a single click (Figure 2A). More sophisticated approaches could include graded ratings—e.g., 5-point responses, like those used by Amazon or Netflix—or separate rating dimensions such as methodological rigor, creativity and innovation, substantive impact, etc., providing users with an immediate snapshot of the strengths and weaknesses of each article.

### Threaded commenting

A key feature of the reddit platform (and many of its precursors, e.g., Slashdot.org) is threaded discussion: users can comment on and rate not only on primary documents (in this case, scientific articles), but also other comments (Figure 2B). This feature is vital to the success of a collaborative filtering platform, as it provides a highly efficient corrective mechanism. For example, it is common on reddit to see one comment's score change dramatically in a span of hours in response to additional comments. This format should translate exceptionally well to the domain of scientific evaluation, where a single user has the potential to raise important concerns that other researchers may have overlooked but can nonetheless appreciate. To encourage authors to engage with other commenters, one might designate verified author comments with a special icon (e.g., Figure 2B, orange), and perhaps provide a small ratings boost to such comments.

### Reputation system

To incentivize users to comment on and review papers (and participate in threaded discussions of those reviews), the evaluation platform should feature a robust reputation system that combines basic features of the reddit system with additional features found in the Stack Exchange platform. The reddit system awards users “karma” points for sharing links and writing comments that are favorably rated by other users; in the context of a scientific evaluation platform, users would receive points based on ratings of their contributed articles on the one hand and their comments and reviews on the other (these commenting and authorship metrics would be kept separate). Each user's reputation and a summary of their contributions would be viewable from the user's public page. Standard filtering and search options would be available, allowing other users to see, e.g., what an individual's top-rated or newest articles or comments are.

### Categorization

Much as reddit features “subreddits” geared toward specific topics and niche interests (e.g., science, cooking, or politics), articles in the database would be organized by topic. A core set of topics could be automatically generated based on keywords (e.g., the National Library of Medicine's Medical Subject Heading [MeSH] ontology); thus, for example, navigating to the /keyword/neuroimaging subdirectory would display a ranked list of all articles tagged with the “neuroimaging” keyword (e.g., Figure 2). Additionally, however, users would be able to create their own custom topics tailored to more specific niches, much as any reddit user currently has the ability to create new subreddits. This two-pronged approach would balance the need for relatively objective ontologies with manually curated sets of articles (where the role of curator would be somewhat similar to that of an editor in the conventional publishing system). An additional benefit of topic-based organization is that articles published in domains with very different citation rates and community sizes could be easily normalized and put on a common metric, much as the reddit front page currently normalizes scores of links submitted to different subreddits.

### Ranking

For any given set of articles retrieved from the database, a ranking algorithm would be used to dynamically order articles on the basis of a combination of quality (an article's aggregate rating in the system), relevance (using a recommendation system akin to Netflix or Amazon's), and recency (newly added articles would receive a boost). By default, the same algorithm would be used for all users (as on reddit). However, as discussed above, allowing users to customize the algorithm used to rank articles and/or weight researchers contributions would greatly increase the utility of the basic platform by enabling individuals or groups with specific goals to filter articles or users more efficiently (e.g., faculty search committees with specific needs could rank candidates based on a customized set of criteria).

### API access

To facilitate community engagement and allow third parties to use evaluation data in creative new ways, a public API should be provided that enables programmatic access to nearly all platform data (with the exception of data where privacy is a potential issue—e.g., individual users' ratings of individual articles).

Importantly, these features need not all be implemented at once. In particular, recommendation systems, customizable ranking algorithms, and a public API, while all desirable, could be added at later stages of implementation once the basic platform was operational. Of course, many other features not mentioned here could also be added later—e.g., social networking features, integration with third-party evaluation metrics (e.g., total-impact.org), a closed-review phase that allows users to solicit reviews privately before an article's public release, and so on.

## Conclusion

In the preface to *On Spirals*, Archimedes amusingly reveals that, on at least one occasion, he deliberately sent his colleagues in Alexandria false theorems, “so that those who claim to discover everything, but produce no proofs of the same, may be confuted as having pretended to discover the impossible” (Bombieri, 2011). This age-old concern with being scooped by other researchers will no doubt be familiar to many contemporary scientists. What's not so easily understandable is why, in an age of preprint servers, recommendation systems, and collaborative filters, we continue to employ publication and evaluation models that allow such concerns to arise so frequently in the first place. While healthy competition between groups may be conducive to scientific progress, delays in the review and publication process are almost certainly not. Inefficiencies in our current evaluation practices are visible at every stage of the process: in the redundancy of writing and re-writing articles in different formats to meet different journals' guidelines; in the difficulty editors face in locating appropriate reviewers; in the opacity and unreliability of the pre-publication review process; in the delays imposed by slow reviews and fixed publication schedules; in limitations on access to published articles; and in the lack of centralized repositories for post-publication evaluation of existing work. Almost without exception, effective technical solutions to these inefficiencies already exist, and are in widespread use on the social web. And yet, almost without exception, the scientific community has ignored such solutions in favor of an antiquated evaluation model that dates back hundreds of years—and in some respects, all the way back to the ancient Greeks.

To take a long view, one might argue that such inefficiencies are not the end of the world; after all, science is a cumulative, self-correcting enterprise (Peirce, 1932; Platt, 1964; Popper, 2002). Given sufficient time, false positives work themselves out of the literature, bad theories are replaced by better ones, and new methods emerge that turn yesterday's tour-de-force analysis into today's routine lab assay. But while the basic truth of this observation isn't in question, it's also clear that all cumulative efforts are not equal; the rate at which we collectively arrive at new scientific discoveries counts for something too. Ideally, we'd like to find cures for diseases, slow the aging process, and build colonies on extra-solar planets sooner rather than later. Since the rate of scientific discovery is closely tied to the rate of dissemination and evaluation of scientific output, the research community has an enormous incentive—and arguably, a moral duty—to improve the efficiency and reliability of the scientific evaluation process. From a utilitarian standpoint, it seems almost certain that even relatively small increases in the rate of scientific publication and evaluation would, compounded over time, have far greater societal benefits than all but a very few original scientific discoveries. We should act accordingly, and not let inertia, lack of imagination, or fear of change prevent us from realizing new models of scientific evaluation that are eminently feasible given present-day technologies.

### Conflict of interest statement

The author declares that the research was conducted in the absence of any commercial or financial relationships that could be construed as a potential conflict of interest.
